# Comparison of the Effectiveness of Nigella Sativa and Vitamin E in Preventing Intra‐abdominal Adhesions

**DOI:** 10.1002/vms3.70446

**Published:** 2025-06-01

**Authors:** Musa Ozekinci, Murat Koc, Latif Emrah Yanmaz, Ozlem Ozmen

**Affiliations:** ^1^ Department of Traditional, Complementary and Integrative Medicine Ankara Yıldırım Beyazıt University Ankara Turkey; ^2^ Department of Surgery Faculty of Veterinary Medicine Burdur Mehmet Akif Ersoy University Burdur Turkey; ^3^ Department of Pathology Faculty of Veterinary Medicine Burdur Mehmet Akif Ersoy University Burdur Turkey

**Keywords:** collagen, inflammation, peritoneum, tissue adhesion, vitamins

## Abstract

**Objective:**

To compare the efficacy of locally applied Nigella sativa (NS) oil and Vitamin E (vit E) in a rat adhesion model.

**Animals:**

Thirty‐six male Wistar rats (90 days old, 240 ± 37 g)

**Methods:**

Animals were assigned to three groups: control (sterile saline), NS oil (10 mg/kg), and vit E (50 mg/kg). After inducing intra‐abdominal adhesion, the treatments were administered intraperitoneally. On day 8, rats were euthanized, and adhesions were scored macroscopically. Levels of IL‐6, TNF‐α, PGE2, TGF‐β1, VEGF, IL‐10 and IL‐1β were measured, and histopathological evaluation and Picro‐Sirius Red staining were performed.

**Results:**

Adhesion scores did not significantly differ between groups (*p* = 0.680). TNF‐α levels were significantly higher in the control group compared to the vit E group (*p* = 0.011), with no significant difference between the NS oil and vit E groups. PGE2 levels differed significantly between the NS oil and vit E groups (*p* = 0.023). IL‐1β, IL‐6, IL‐10, TGF‐β1 and VEGF levels did not show significant differences (*p* > 0.05). Histopathology revealed reduced fibrosis and inflammation in the vit E group compared to NS oil (*p* < 0.001). Picro‐Sirius Red staining showed improved collagen maturation in the vit E group, followed by NS oil, with the control group exhibiting less mature collagen.

**Conclusion:**

Both treatments showed efficacy, but vit E was superior in reducing inflammation and fibrosis and enhancing collagen maturation.

## Graphical abstract

In a rat model of intra‐abdominal adhesions, Vitamin E (vit E) and Nigella sativa (NS) oil were compared for their therapeutic effects.

While both reduced inflammation and fibrosis, vit E demonstrated superior efficacy, significantly lowering TNF‐α levels, reducing fibrosis, and enhancing collagen maturation. This suggests Vitamin E is more effective in preventing post‐surgical adhesions.

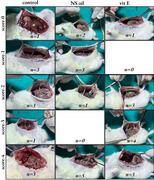

## 1 Introduction

Intra‐abdominal adhesions are pathological fibrous bands that may form between abdominal organs as a consequence of surgical procedures. These adhesions are primarily linked to damage to mesothelial cells, leading to fibrin deposition and inflammatory reactions. (Irkorucu et al. 2009) The balance between fibrin production and fibrinolysis is critical in determining adhesion formation. (Zarzycki et al., 2021) Adhesions stemming from peritoneal inflammation can adversely affect quality of life, causing complications such as difficulty during subsequent surgeries and persistent abdominal or pelvic discomfort. (Wang et al., 2022) Despite research advancements, the precise mechanisms driving adhesion formation remain unclear, and no universally effective prevention strategy has been identified. (Butureanu and Butureanu 2014) Pharmacological methods that focus on reducing inflammation, modulating coagulation, and enhancing fibrinolysis have shown potential. (Hu et al., 2021, Stratakis et al., 2023)

Various antioxidant compounds, particularly those derived from herbal medicines, have been investigated in animal models for their potential to prevent postoperative peritoneal adhesions. (Karatas and Ozlu 2014) Salvia miltiorrhiza extract, recognised for its potent antioxidant and anti‐inflammatory properties, significantly reduced macroscopic and microscopic adhesion scores, oxidative stress markers, and proinflammatory cytokines in a rat model. (Raisi et al., 2021) Similarly, Rosmarinus officinalis extract and its active constituent, rosmarinic acid, demonstrated the ability to decrease levels of inflammatory cytokines, fibrosis markers, oxidative stress and the overall severity of adhesions. (Kakanezhadi et al. 2022, Roohbakhsh et al. 2020) A recent review further emphasised that both traditional herbal remedies and modern pharmacological agents target key pathways involved in adhesion formation, such as fibrin deposition, inflammation and angiogenesis. However, their clinical applicability remains limited and requires further investigation. (Soltany 2021)

Vitamin E (vit E) refers to a group of compounds collectively known as tocopherols and tocotrienols, which are found in various natural products of both plant and animal origins. (Gamna and Spriano 2021) As a lipid‐soluble vitamin integral to cell membranes, vit E exhibits notable antioxidant, anti‐inflammatory, anticoagulant and antifibrotic effects. (Rizvi et al., 2014) Its antioxidant role includes neutralising free radicals and inhibiting enzymatic and non‐enzymatic lipid peroxidation, thereby preserving cellular integrity. (Yildiz et al. 2011) Studies have demonstrated the effectiveness of intraperitoneally administered vit E in minimising the formation of intra‐abdominal adhesions. (Portilla et al. 2004, Sudirman et al. 2022)

Nigella sativa (NS), commonly known as black seed, is a herbaceous plant belonging to the Ranunculaceae family and is widely distributed across regions such as North Africa, the Middle East, Europe and Asia. (Dalli et al., 2021) NS exhibits a wide range of pharmacological properties, including antioxidant, anti‐inflammatory, antihistamine, antimicrobial and immunomodulatory effects. (Sahbaz et al. 2014) It has been utilised to manage conditions such as cardiovascular and gastrointestinal disorders, respiratory diseases, hypertension, dyslipidaemia, diabetes, and various types of cancer. (Pop et al., 2020) NS has also been explored for treating neurological disorders, hyperlipidaemia, obesity, rheumatoid arthritis, thyroid dysfunction, hepatitis and infertility. (Mashayekhi‐Sardoo et al. 2020) Studies suggest that NS oil application after surgical injury significantly reduces adhesion formation by minimising macroscopic adhesion, fibrosis, inflammation and vascularisation. (Sahbaz et al. 2014)

Based on a literature review, no studies to date have directly compared the effects of NS and vit E in preventing intra‐abdominal adhesions. This study was aimed to evaluate and compare the efficacy of locally applied NS and vit E in an experimental rat adhesion model. We hypothesise that NS will demonstrate greater effectiveness than vit E in reducing intra‐abdominal adhesion formation.

## 2 Materials and Methods

A total of 36 male Wistar rats, aged 90 days and weighing 240 ± 37 g, were used. All rats were obtained from the XXX University Animal Experiment Centre. All procedures were conducted in accordance with guidelines suggested by the recommendations of the National Research Council's Guide for the Care and Use of Laboratory Animals and with the approval of the XXX Local Board of Ethics Committee for Animal Experiments (no.: 2023/1118). Rats were housed six per cage prior to the initialisation of the experiments. The experimental room was maintained at a humidity range of 40–60%, a temperature of 22 ± 2°C, and an illumination of a 12:12 h light/dark cycle. All rats were nourished with a standard laboratory‐balanced commercial diet with free access to water during the study.

Following the creation of the intra‐abdominal adhesion model, each rat was randomly assigned to one of three groups, with each group comprising 12 animals. The groups were categorised based on the intraperitoneal treatment administered: the control group received 1 mL sterile saline (0.9% NaCl, Biofleks, Osel, Istanbul, Turkey), the NS oil group received 10 mg/kg NS oil (Nu‐Ka Defne Essencia, Alanya, Turkey), and the vit E group received 50 mg/kg vit E (Evin, Farmalas, Istanbul, Turkey). The doses of NS oil and vit E were selected based on previous studies assessing their effectiveness in preventing postoperative adhesions. (Atilgan et al. 2015, Bozdag et al. 2015) In the NS oil and vitamin E groups, sterile saline was added to adjust the total injection volume to 1 mL. To ensure a uniform suspension, the mixtures were thoroughly mixed immediately prior to administration.

### 2.1 Surgery

All animals were anaesthetised via intramuscular administration of a combination of 50 mg/kg ketamine hydrochloride (Keta‐Control 50 mg/kg, 10 mL, Doga Ilac, Istanbul, Turkey) and 5 mg/kg xylazine hydrochloride (Xyla 5 mg/kg, Intercheima, Venray, Holland) delivered in the same syringe. Following anaesthesia, the surgical area was shaved, and a 10% povidone‐iodine solution was applied for antisepsis. A 3 cm midline incision was made, and four peritoneal buttons were created on the left side of the parietal peritoneum using 3‐0 Vicryl sutures in a chain distribution, as described previously. (Okur et al. 2024) Each suture enclosed approximately 1 cm^2^ of parietal peritoneum, with the suture diameter measuring approximately 0.2 cm. After the intraperitoneal treatment application, the linea alba, muscle, and subcutaneous tissues were closed with a simple continuous suture pattern using 4/0 absorbable polydioxanone, and the skin was closed with a simple interrupted pattern using 4/0 polypropylene.

### 2.2 Analytical Methods for NS Oil

The quantification and separation of thymoquinone in NS oil were performed using high‐performance liquid chromatography (HPLC) with a reverse phase C‐18 column and an Agilent 1260 series HPLC‐UV system. A calibration curve for thymoquinone quantification was established within the concentration range of 5–100 µg/ml, and the results were analysed using the calibration equations. Volatile components in the NS oil were identified using solid‐phase microextraction gas chromatography‐mass spectrometry. The thymoquinone concentration in the NS oil was determined to be 11.94 ± 0.57 mg/ml, with thymoquinone accounting for 53.78% relative abundance of the volatile components.

### 2.3 Macroscopic Evaluation

On day 8, all rats were euthanized by cervical dislocation under anaesthesia. To expose the abdominal cavity, a linear incision was performed. The extent and severity of intra‐abdominal adhesions were evaluated by two independent researchers, who were blinded to the treatments, using the adhesion scoring system described by Nair et al. (1974) Adhesion grades were defined as follows: grade 0, no adhesions; grade 1, thin and filmy adhesions; grade 2, thick adhesions confined to a small area; grade 3, extensive thick adhesions; and grade 4, extensive thick adhesions with visceral organs attached to the abdominal wall. (Rakhshandeh et al. 2023)

### 2.4 Biochemical Evaluation

Peritoneal tissue samples were collected from the peritoneal surface adjacent to the adhesion site in each rat, immediately transferred to lithium heparin tubes, and centrifuged at 3000 rpm for 10 min at +4°C. The supernatant was separated, and tissue samples were stored at ‐20°C until biochemical analyses were performed. The levels of Interleukin‐6 (IL‐6, E0135Ra), Tumour Necrosis Factor Alpha (TNF‐α, E0764Ra), Prostaglandin E2 (PGE2, E0504Ra), Transforming Growth Factor Beta 1 (TGF‐β1, E1688Ra), Vascular Endothelial Growth Factor (VEGF, E0659Ra), Interleukin‐10 (IL‐10, E0108Ra), and Interleukin‐1 Beta (IL‐1β, E0119Ra) were measured using commercially available ELISA kits (Bioassay Technology Laboratory, Shanghai, China). The analysis was conducted using a spectrophotometer (Allsheng AMR‐100, China), following the manufacturer's protocols.

### 2.5 Histopathological Evaluation

The tissue samples collected at the conclusion of the assessment were preserved in a 10% formaldehyde solution for a duration of 48 h. Subsequently, they were encased in paraffin blocks using standard tissue processing protocols. Sections of 5 µm in thickness were obtained from each block. The samples were then processed for histopathologic evaluation and stained with haematoxylin‐eosin. Finally, the samples were analysed using light microscopy (Olympus CX21, Tokyo, Japan). Histopathological findings were graded based on fibrosis scoring (0–3), inflammation scoring (0–3) and vascular proliferation scoring (0–3), as described earlier. (Hooker et al. 1999) A separate series of sections obtained on standard slides was used to evaluate connective tissue development through Picro‐Sirius Red staining. The manufacturer's staining protocol was followed using the Picro Sirius Red Stain Kit (ab150681‐Picro Sirius Red Stain Kit, Abcam, Cambridge, UK). Briefly, deparaffinised and rehydrated sections were completely covered with Picro‐Sirius Red solution and incubated for 30 minutes. Following incubation, the sections were rinsed twice with acetic acid solution. After the staining process, the sections were dehydrated, cleared with xylene, and mounted with Entellan. The prepared slides were then examined under a light microscope. (Rittié 2017)

### 2.6 Statistical Analysis

A power analysis was conducted to determine the minimum number of animals required per group for the study (PS‐Power and Sample Size Calculation, Version 3.1.2, Vanderbilt University, TN, USA). The analysis was designed with a Type I error (α) of 0.05 and a power (1−β) of 0.90. Based on this analysis, a minimum of 12 animals per group was required to detect a statistically significant difference of 1.5 in adhesion scores (standard deviation [SD] ± 1) evaluated macroscopically. The power calculation was performed based on a previous study investigating adhesion prevention. (Moradi et al. 2023)

Statistical analysis was performed using SPSS software (Version 25.0, IBM Corp., Chicago, IL, USA). The Shapiro–Wilk test was used to assess the normality of data distribution, while Levene's test was applied to evaluate the homogeneity of variances. Data with a normal distribution (IL‐1β, IL‐6, IL‐10, TGF‐β1, TNF‐α and VEGF) were analysed using one‐way analysis of variance, followed by post hoc Tukey's test to identify intergroup differences. Non‐normally distributed data (adhesion, fibrosis, inflammation, and vascular proliferation scores) were analysed using the Kruskal–Wallis test. When the Kruskal–Wallis test indicated significant differences, post hoc Dunn's test was performed to determine specific group differences. Statistical significance was set at 𝑝 < 0.05. Normally distributed data are presented as mean ± SD, while non‐normally distributed data are reported as median (range [min–max]).

## 3 Results

**FIGURE 1:**
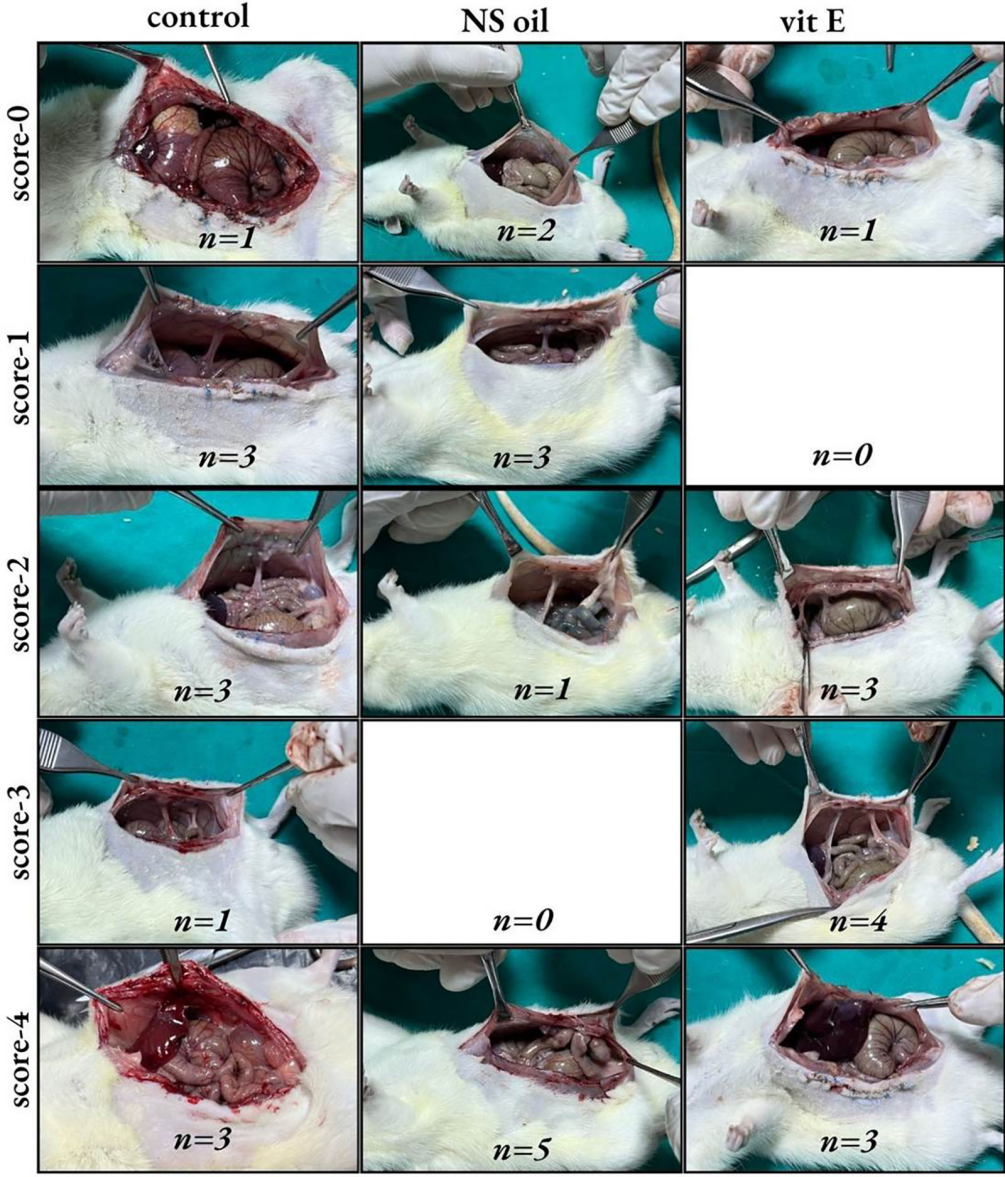
Macroscopic evaluation of adhesion scores in the control, vitamin E (vit E) and Nigella sativa **(NS)** oil groups after 7 days.

**FIGURE 2:**
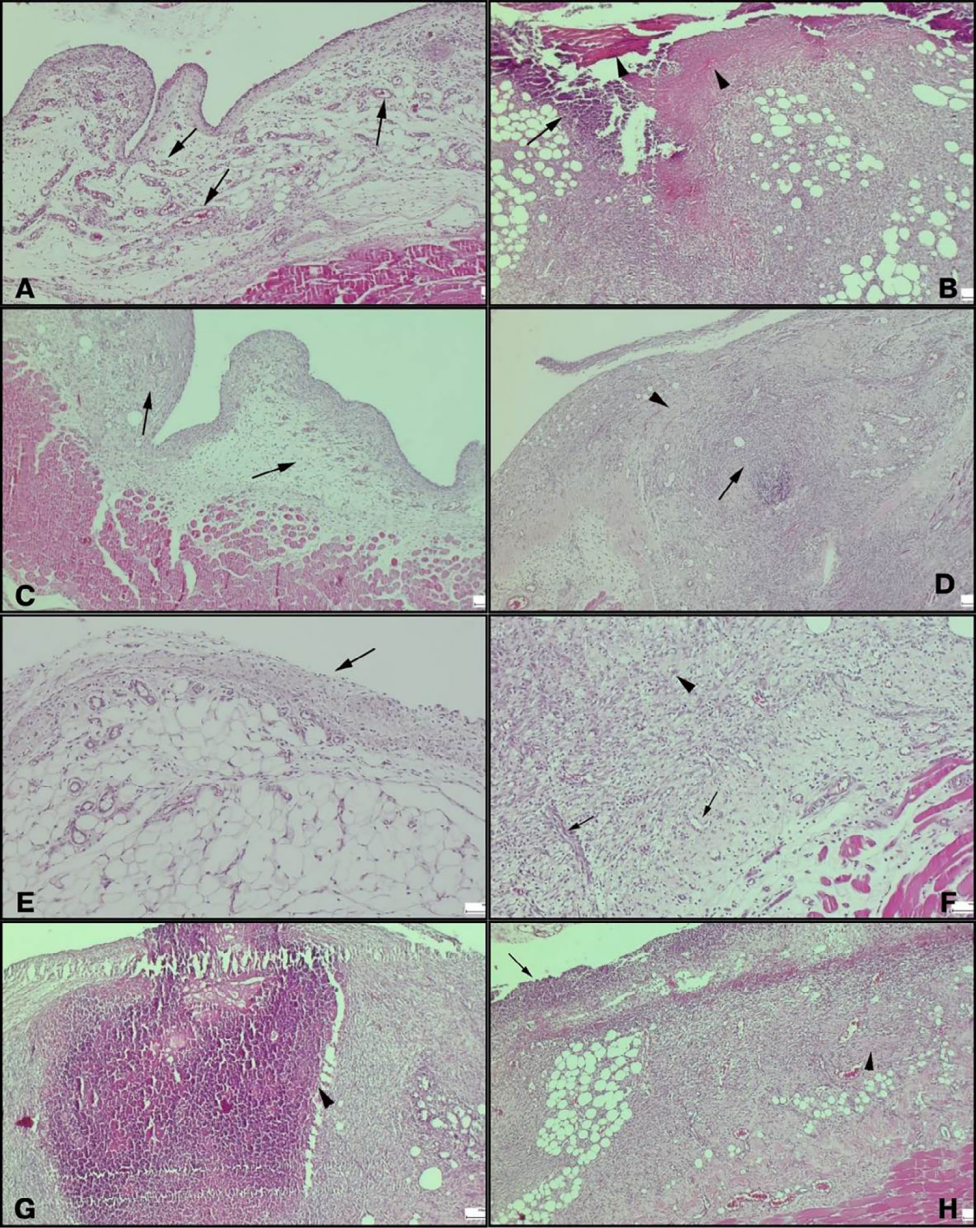
Histopathological evaluation of control, vitamin E (vit E), and Nigella sativa (NS) oil groups on day 7: **(A)** Severe oedema, moderate vascularisation (arrows), and mild inflammation in a rat from the control group, (**B)** Peritoneal reaction in a rat from the control group with severe peritonitis. Prominent necrotic masses (arrowheads) and inflammatory reaction (arrow), **(C)** Mild peritonitis and slight increase in connective tissue in a rat from the vit E group, **(D)** Severe peritonitis with moderate inflammation (arrow) and increased connective tissue (arrowhead) in a rat from the vit E group, **(E)** Mesothelial cell exfoliation (arrow) in a region with increased vascularisation in the vit E group, **(F)** Severe inflammation (arrow) and prominent fibrosis (arrowhead) in a rat from the NS oil group. Newly formed blood vessels (arrow) and widespread fibrosis (arrowhead), **(G)** Severe abscess (arrowhead) and fibrosis in a rat from the NS oil group and **(H)** Severe peritonitis characterised by intense inflammation (arrow) and severe fibrosis (arrowhead) in a rat from the NS oil group. A, B, C, D, G and H: Scale bar = 200 µm; E and F: Scale bar = 100 µm.

**FIGURE 3:**
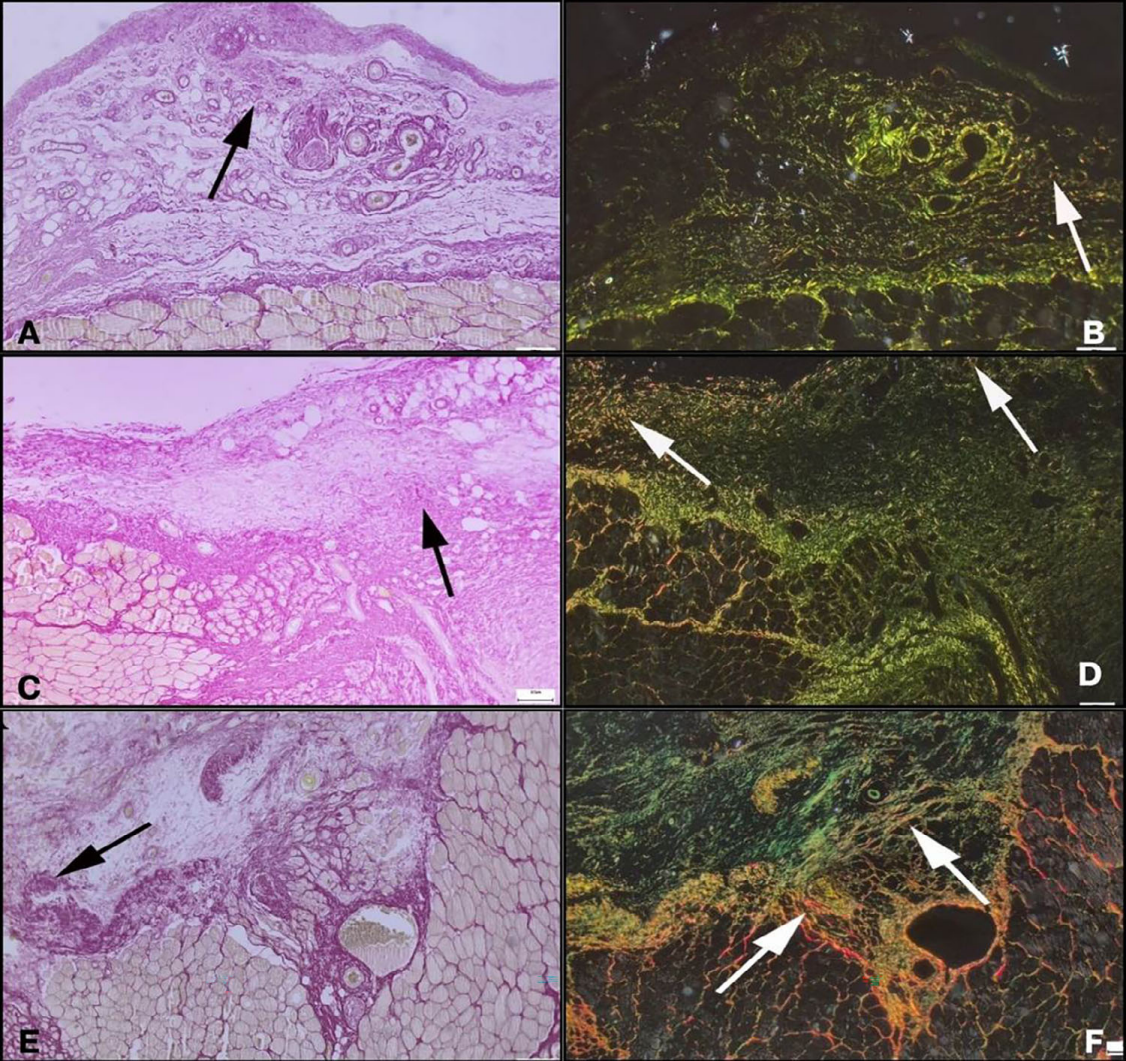
Picro Sirius Red evaluation of control, Vitamin E (vit E), and Nigella sativa (NS) oil groups after 7 days: **(A)** Appearance of increased connective tissue (arrow) from the control group, **(B)** The same area under polarized light filter shows prominent green fluorescence indicating immature Type III collagen and slightly increased red staining indicating mature Type I collagen (arrows), **(C)** Appearance of increased connective tissue development (arrow) from the NS oil group, **(D)** The same area under polarized light filter shows intense green fluorescence indicating immature Type III collagen and slightly increased red staining indicating mature Type I collagen (arrows), **(E)** Appearance of increased connective tissue development (arrow) from the vit E group and **(F)** The same area under polarized light filter shows widespread green fluorescence indicating immature Type III collagen along with a prominent increase in red staining indicating mature Type I collagen (arrows). Scale bars = 50 *µm*.

A total of three animals died during the experiment, one from each group. The remaining animals completed the study without any complications. Adhesion scores for the control group (2, range 0–4), NS oil group (2, range 0–4), and vit E group (3, range 0–4) did not differ significantly among the groups (*p* = 0.680) (Figure 1). TNF‐α levels were significantly higher in the control group (237 ± 26.5 ng/L) compared to the vit E group (203 ± 27 ng/L, *p* = 0.011) but not significantly different from the NS oil group (215 ± 22.2 ng/L, *p* = 0.118). PGE2 levels showed a significant difference between the NS oil and vit E groups (*p* = 0.023). IL‐1β, IL‐6, IL‐10, TGF‐β1 and VEGF levels did not differ significantly among the groups (*p* > 0.05 for all; Table 1). The vit E group demonstrated reduced fibrosis and inflammation compared to the NS oil group (*p* < 0.001). However, no significant differences were found in vascular proliferation among the groups (*p* = 0.362; Table 2) (Figure 2). Picro‐Sirius Red staining revealed extensive connective tissue development in the control group, primarily consisting of immature type III collagen, with early indications of mature type I collagen formation. Collagen maturation was moderately more pronounced in the NS oil group compared to the control, while the vit E group exhibited a significantly higher degree of collagen maturation (Figure 3).

**TABLE 1:** Comparison of IL‐1β, IL‐6, IL‐10, PGE2, TGF‐β1, TNF‐α and VEGF levels among the control (C), vitamin E (vit E), and Nigella sativa oil (NS) oil groups. Data are presented as mean ± standard deviation.

	Biochemical Parameters						
Groups	IL‐1β (ng/ml)	IL‐6 (ng/L)	IL‐10 (pg/ml)	PGE2 (ng/ml)	TGF‐β1 (ng/L)	TNF‐α (ng/L)	VEGF (ng/L)
C	14.5 ± 1.6^a^	9.8 ±1.2	152 ± 14^a^	2.6 ± 0.3^a^	414 ± 42^a^	237 ± 26^a^	571 ± 60
NS	12.6 ± 1.6^b^	8.1 ± 0.8	138 ± 15^ab^	2.6 ± 0.4^a^	361 ± 44^b^	215 ± 22.2^ab^	535 ± 69
Vit E	11.7 ± 1.7^b^	8.2 ± 0.9	129 ± 21^b^	2.2 ± 0.3^b^	357 ± 18^b^	203 ± 27^b^	493 ± 97
*p*‐ value	0.002	0.147	0.0024	0.017	0.019	0.014	0.071

Letters with different superscripts in the same column indicate significant differences between groups (*p* < 0.05).

**TABLE 2:** Comparison of fibrosis, inflammation and vascular proliferation scores among the control (C), vitamin E (vit E), and Nigella sativa (NS) oil groups. Data are presented as mean ± standard deviation.

Groups	Histopathological parameters		
	Fibrosis	Inflammation	Vascular proliferation
C	2 (1–2)^a^	2 (1–3)^a^	2 (1–2)
NS	3 (2–3)^b^	3 (2–3)^b^	2 (1–2)
Vit E	1 (1–2)^a^	2 (1–2)^a^	2 (1–2)
*p*‐value	< 0.001	<0.001	0.362

Letters with different superscripts in the same column indicate significant differences between groups (*p* < 0.05).

## 4 Discussion

This study aimed to evaluate and compare the efficacy of NS oil and vit E in preventing intra‐abdominal adhesions in an experimental rat model. Our results demonstrated that both treatments were effective in reducing adhesion formation to some extent; however, Vit E exhibited superior anti‐inflammatory and antifibrotic effects compared to NS oil. Specifically, Vit E significantly reduced fibrosis and inflammation scores compared to both NS oil and the saline group. Biochemical analysis supported these findings, showing lower levels of pro‐inflammatory cytokines such as TNF‐α and PGE2 in the vit E group. Histopathological findings also revealed better collagen maturation in the vit E group. These results support the hypothesis that NS oil reduces adhesion formation; however, the hypothesis that NS oil would be more effective than vit E was rejected.

Vit E, a fat‐soluble vitamin present in all cell membranes, has been studied for its potential to prevent postoperative adhesions. Previous research has highlighted its antioxidant properties, as it scavenges free radicals and protects cell membranes from oxidative degradation. It also acts as an anti‐inflammatory agent by inhibiting COX‐2 and the arachidonic acid pathway, reducing levels of pro‐inflammatory mediators such as PGE2 and TNF‐α. Additionally, vit E decreases TGF‐β activity, a potent fibrosis inducer, which may explain its role in reducing fibrotic band persistence and mitigating adhesion formation. (Arung et al. 2011, Corrales et al. 2008) These mechanisms contribute to the prevention of postoperative adhesions, as seen in our study where vit E reduced both fibrosis and inflammation, which aligns with findings from earlier studies. (Sudirman et al., 2022; Yetkin et al., 2009)

Picro‐Sirius Red analysis revealed that vit E reduced VEGF and collagen type I expression in the presence of adhesions, consistent with a previous study. (Atilgan et al. 2015) These findings suggest that vit E may reduce neovascularisation and collagen deposition, potentially by modulating pro‐inflammatory and pro‐fibrotic factors. Despite the promising trends observed, the statistical significance was not achieved in all parameters, indicating that further studies with optimised dosages and methods are necessary to validate these effects. The enhanced collagen maturation observed with Picro‐Sirius Red staining further underscores vit E's potential to encourage structured tissue repair. These findings are consistent with prior research demonstrating the efficacy of intraperitoneal vit E in reducing adhesion formation. (Portilla et al. 2004, Sudirman et al. 2022)

In contrast, the NS oil group exhibited a different response. While NS oil demonstrated a significant reduction in macroscopic adhesions, fibrosis and vascularisation scores, it showed less efficacy than vit E in reducing cytokine levels such as TNF‐α, IL‐6, and VEGF. The observed reduction in collagen maturation was also less pronounced in the NS oil group compared to vit E. This suggests that while NS oil may reduce adhesion formation to some extent, it does not match the anti‐inflammatory and antifibrotic effects seen with vit E. Our study also revealed peritonitis in the NS oil group, a complication not observed in the other groups. This may be attributed to the saline solution used in the study, which has been shown to induce oxidative stress and disrupt the fibrinolytic properties of mesothelial cells, potentially exacerbating inflammation. (Polubinska et al. 2008)

NS oil, rich in thymoquinone, also exhibits notable antioxidant and anti‐inflammatory properties, supporting the body's defence against free radicals. (Dalli et al., 2021) NS oil's potential to modulate fibrinolysis and thrombus formation suggests its viability as an anti‐adhesion agent. (Sahbaz et al. 2014) Studies have demonstrated that NS oil reduces the severity of adhesions by inhibiting COX and 5‐LO pathways, highlighting its ability to diminish the production of inflammatory mediators. (Mashayekhi‐Sardoo et al. 2020) Furthermore, the viscoelastic nature of NS oil may provide a physical barrier between peritoneal surfaces, reducing trauma‐induced adhesion formation. However, in our study, NS oil showed limited effects on reducing cytokine levels like TNF‐α, IL‐6 and VEGF compared to vit E, which may explain its comparatively lower efficacy in adhesion prevention. Nevertheless, the moderate improvement in collagen maturation observed with NS oil underscores its potential to support tissue repair.

Biochemical analyses supported the observed differences between vit E and NS oil. Vit E reduced levels of pro‐inflammatory cytokines such as TNF‐α and PGE2 more effectively than NS oil, aligning with its known anti‐inflammatory properties. The suppression of TNF‐α and PGE2 reflects vit E's ability to reduce macrophage and neutrophil activation during the early stages of inflammation. (Fredriksson et al. 2014) Interestingly, although both vit E and NS oil reduced IL‐1β and IL‐6 levels, the differences between the two were not statistically significant. However, vit E was notably more effective at reducing IL‐10 levels compared to saline. While IL‐10 is typically associated with anti‐inflammatory effects, this unexpected reduction may reflect a complex modulation of the inflammatory response by vit E. This suggests that the reduction in adhesion severity may be mediated through mechanisms other than IL‐10, potentially involving the suppression of other pro‐inflammatory cytokines such as TNF‐α and PGE2.

Our findings also provide insight into the molecular mechanisms underlying adhesion formation. Elevated levels of pro‐inflammatory cytokines like IL‐6, TNF‐α and PGE2 contribute to fibrin formation and the subsequent development of adhesions. (Moradi et al. 2023, Wang et al. 2022) The reduction in IL‐6 and VEGF levels in the vit E group, although not statistically significant, suggests that vit E may attenuate the inflammatory and angiogenic pathways responsible for adhesion formation.

Several limitations should be acknowledged in this study. The use of a single dose for each treatment restricts our ability to evaluate dose‐dependent effects. Additionally, while histopathological and biochemical analyses provided valuable insights, further studies incorporating molecular assays are needed to clarify the specific pathways involved in adhesion prevention. Furthermore, the potential for synergistic effects between vit E and NS oil warrants further exploration, as combining these substances may optimise their therapeutic benefits.

In conclusion, our study provides valuable insights into the potential therapeutic effects of vit E and NS oil in reducing postoperative adhesions. While both treatments exhibited some efficacy, vit E demonstrated superior anti‐inflammatory and antifibrotic effects, as well as enhanced collagen maturation. These findings suggest that vit E may be a more effective pharmacological agent for reducing postoperative adhesions, with potential clinical applications. Further studies are warranted to optimise its use and explore its combination with NS oil to maximise therapeutic benefits.

## Author Contributions

**Musa Ozekinci**: study design, data management, data interpretation and preparation of the manuscript. **Murat Koc**: study design and supervisor. **Latif Emrah Yanmaz**: data management, data interpretation, statistical analysis and revision of the manuscript. **Ozlem Ozmen**: histopathological analysis and data interpretation.

## Ethics Statement

The study protocol was carried out after approval by the Burdur Mehmet Akif Ersoy Local Ethics Committee (Decision no: 2023/1118).

## Conflicts of Interest

The authors declare no conflicts of interest.

### Peer Review

The peer review history for this article is available at https://www.webofscience.com/api/gateway/wos/peer‐review/10.1002/vms3.70446.

## Data Availability

Data is available on request from the authors.
